# Optimization of Vacuum Frying for Black Glutinous Rice Crackers

**DOI:** 10.3390/foods15122239

**Published:** 2026-06-21

**Authors:** Anh Hoang Tuyet Nguyen, Nantawan Therdthai, Chonnikarn Srikanlaya

**Affiliations:** Department of Product Development, Faculty of Agro-Industry, Kasetsart University, Bangkok 10900, Thailand; anhhoangtuyet.n@ku.th (A.H.T.N.); chonnikarn.srik@ku.th (C.S.)

**Keywords:** vacuum frying, black glutinous rice, model, antioxidant, optimization

## Abstract

This study aimed to optimize vacuum frying parameters, frying temperature (80–120 °C) and frying time (10–20 min), using response surface methodology (RSM) to maximize the quality of rice crackers from black glutinous rice. Vacuum frying temperature and time had no significant (*p* > 0.05) effect on protein, fiber, total anthocyanin content, and total flavonoid content. An increase in frying temperature increased the expansion ratio and total phenolic content (TPC), while decreasing bulk density and DPPH. Extending frying time significantly (*p* ≤ 0.05) increased fat content. Increasing both frying temperature and time reduced hardness, moisture, and water activity, and significantly changed color. These trends were evaluated using regression models with R^2^ values ranging from 0.858 to 0.999. Based on the developed models, the optimal condition was estimated at approximately 110 °C for 10 min, graphically predicting rice crackers with 23.32%db fat, hardness of 4.83 N, and TPC of 2.63 mg GAE/g. Compared with atmospheric frying (160 °C, 10 min), the optimal vacuum frying condition (110 °C, 10 min) reduced fat by 36.16%, decreased hardness by 68.65%, and increased TPC by 95.49%, suggesting that vacuum frying can produce black glutinous rice crackers with lower fat, higher antioxidant compounds, and greater crispiness under these specific parameters.

## 1. Introduction

In recent years, cereal-based foods have represented a fundamental component of human diets in a variety of forms, including crackers, serving as a staple source of energy and nutrients. Owing to their extended shelf life and desirable sensory qualities, the consumption of crackers has increased, making them one of the most popular snack products worldwide [1]. Traditionally, rice crackers are produced via atmospheric deep-fat frying, a method widely used to prepare snacks such as potato chips and tortilla chips, nuts, and crackers [2]. However, a major drawback of this conventional method is high oil retention, which makes the crackers oily and significantly shortens their shelf life. Conventional atmospheric frying involves submerging foods in oil at a temperature ranging from 120 to 200 °C, with the most common range being 170 to 190 °C. While these high temperatures enhance the surface color and mechanical characteristics of fried foods [3], producing fried snacks with reduced oil content, high nutritional value, enhanced crispiness, and improved sensory acceptability has become a primary focus of the food industry.

Vacuum frying technology offers a distinct advantage by allowing heat-sensitive materials to be processed while retaining their natural color, flavor, and nutrition, all while minimizing oil uptake due to the lower operating temperatures under negative pressure. According to Lee and Demirci [4], a vacuum fryer consists of five major components: a vacuum pump, a frying container, a heating element, a condenser, and an oil-removal system. Under vacuum conditions, the boiling point of water decreases, which facilitates effective water removal at lower temperatures [5]. Additionally, due to the controlled oxygen levels and lowered thermal stress throughout the operation, vacuum frying exerts minimal negative impact on the oil quality, reduces acrylamide formation, and preserves nutritional stability [6]. Consequently, vacuum-fried products (such as apples, carrots, sweet potatoes, cassava, and fish) often contain 25–60% less oil than their atmospheric-fried counterparts. Previous studies have demonstrated that vacuum frying conducted at pressures of 6–25 kPa enables operating temperatures of 80–120 °C, thereby reducing oxidation and excessive Maillard reactions [7,8,9].

Black glutinous rice is a pigmented rice grain, cultivated mainly in Asian countries with warm and humid climates, particularly within Southeast, East, and South Asia [10]. The Leum Pua glutinous rice variety is recognized as an exotic indigenous Thai cultivar valued for its exceptional anthocyanin density and associated health benefits, including antioxidant, anti-cancer, anti-inflammatory, anti-diabetic, and cholesterol-lowering properties [11], highlighting its strong potential as a functional food ingredient. These health-promoting attributes are primarily driven by a rich profile of antioxidant compounds native to the pigmented grain matrix, within which approximately 14 phenolic compounds have been identified. Nevertheless, a substantial fraction of these compounds is bound to structural components of the plant cell wall, such as cellulose, hemicellulose, structural proteins, pectin, and lignin, which inherently limits their release and subsequent bioavailability [12]. Furthermore, flavonoids, a crucial phytochemical group in the black glutinous rice, generally remain stable at temperatures below 80 °C, with partial decomposition initiating between 90 and 100 °C [13]. The color of this grain varies from deep purple to brownish-red in its outer layers due to the accumulation of natural anthocyanins. These water-soluble flavonoids are highly sensitive to pH, temperature, light, oxygen, and metal ions; their degradation is initially observed at 50–60 °C and accelerates exponentially at higher thermal levels [14,15]. Indeed, the literature reports up to an 80% loss of anthocyanins in pigmented rice after cooking, underscoring their high heat sensitivity relative to broader phenolic fractions, which experience a lower loss of approximately 54% [16].

Driven by these health benefits, black glutinous rice has been incorporated into various food products, such as pasta, Thai coconut pancake [17], wine, germinated rice drink, cake [18], and fermented rice cake [19]. Previous studies demonstrated that incorporating higher proportions of black glutinous rice into food formulations improved the preservation of bioactive compounds, contributed to enhanced nutritional value, and health-promoting properties [12]. However, its application in the development of rice crackers characterized by low oil uptake and improved retention of antioxidant compounds has not yet been reported. Therefore, this study aimed to investigate the effects of vacuum frying temperature and time on the physical quality of black glutinous rice crackers and the preservation of their native bioactive compounds (phenolics, flavonoids, and anthocyanins), ultimately optimizing the frying condition to minimize fat content and hardness, without compromising other vital quality attributes.

## 2. Materials and Methods

### 2.1. Materials

Black glutinous rice (*Oryza sativa* L. cv. *Leum Pua*) was purchased from Thai Food Industry (1964) Co., Ltd. (Nonthaburi, Thailand). Palm oil (SD Guthrie International Morakot Public Company Limited, Samut Prakan, Thailand) was obtained from a local supermarket in Thailand. According to the nutritional label, the fatty acid profile of the palm oil consisted of 42.28% saturated, 47.18% monounsaturated (omega-9), and 10.54% polyunsaturated fatty acids (comprising 10.33% omega-6 and 0.21% omega-3), with 0 g trans fat and 0 mg cholesterol.

### 2.2. Preparation of Black Glutinous Rice Crackers

#### 2.2.1. Preparation of Rice Samples Before Frying

The preparation of cooked black glutinous rice was carried out based on the method of Keeratipibul et al. [20] with slight modifications. Black glutinous rice (1000 g) was soaked in distilled water (1:1.5 *w*/*v*) for 16–18 h, drained, and steamed at 100 °C for 120 min. The cooked rice (100 g/batch) was then blended, sheeted to 2 mm thickness, and cut into strips (10 mm × 100 mm; ~5 g/piece). The samples were subsequently fried using a deep-fat fryer and a vacuum fryer according to the experimental design.

#### 2.2.2. Deep Fat Frying of Black Glutinous Rice Crackers

Cooked black glutinous rice (100 g) was fried in a deep-fat fryer (Family Fry F407 CZ, De’Longhi, Treviso, Italy) using 4 L of palm oil. The oil was preheated to the set temperature, and frying was carried out at 160 °C for 10 min based on preliminary results. After frying, the samples were shaken to remove excess surface oil and cooled to ambient temperature. The rice crackers were then packed in aluminum foil bags for further analysis.

#### 2.2.3. Vacuum Frying of Black Glutinous Rice Crackers

The vacuum frying experiment was conducted using a full-factorial design with two factors: frying temperature (80, 100, and 120 °C) and frying time (10, 15, and 20 min). A total of nine treatments (3^2^) were performed using a vacuum fryer (VF-20P, Owner Foods Machinery Co., Ltd., Bangkok, Thailand) operated at a vacuum gauge pressure of 93.33 kPa (corresponding to an absolute pressure of approximately 8 kPa). The resulting black glutinous rice crackers were packed in aluminum foil bags for further analysis.

### 2.3. Physical Quality Determination of Black Glutinous Rice Crackers

#### 2.3.1. Determination of Cooking Yields

The cooking yields were determined based on the ratio of the rice cracker weight before and after frying, using Equation (1).

(1) $$Cooking\;yield\;(\%)=\frac{Weight\;after\;frying}{Weight\;before\;frying}\times 100$$

#### 2.3.2. Determination of Expansion Ratio, Bulk Density, and Water Activity

The thickness of rice crackers before and after frying was measured using a Vernier caliper. Ten samples were analyzed for each frying condition. The expansion ratio was calculated from the thickness before and after frying. For bulk density, the weight of rice crackers in a fixed volume was measured, and bulk density was calculated as weight per unit volume. Water activity (A_w_) was determined using a water activity meter (Novasina Model MS1, Lachen, Switzerland) at 25 °C.

#### 2.3.3. Determination of Color

The surface color of rice crackers was measured using a spectrophotometer (CM-5, Konica Minolta, Tokyo, Japan) in the CIE Lab* system, where L*, a*, and b* represent lightness, redness, and yellowness, respectively. Measurements were conducted under D65 illuminant with a 10° observer. Color difference (ΔE) was calculated using Equation (2).

(2) $$∆E=\sqrt{{{\left({L_{0}}^{*}-L^{*}\right)}^{2}+({a_{0}}^{*}-a^{*})}^{2}+{\left({b_{0}}^{*}-b^{*}\right)}^{2}}$$

where ${L_{0}}^{*}$, ${a_{0}}^{*}$, and ${b_{0}}^{*}$ are the mean lightness, redness, and yellowness values of black glutinous rice before frying, while L*, a*, and b* are the corresponding values after frying.

#### 2.3.4. Determination of Texture

The samples were analyzed using a three-point bending rig (HDP/3PB) attached to a texture analyzer (TA.XT.plus, Stable Micro Systems, Godalming, UK) with a 50 kg load cell. Probe speeds were set at 1 mm/s. Hardness was defined as the maximum force required to break the rice cracker (N).

#### 2.3.5. Determination of Microstructure

The cross-sectional and surface microstructure of black glutinous rice crackers was examined by SEM (SU-3800, Hitachi, Tokyo, Japan) [21]. All samples were dehydrated by freeze-drying and superficially defatted in petroleum ether for 24 h. Then, the samples were gold–palladium-coated and observed at 10 kV under 30× and 100× magnifications using a sputter coater (SC7620, Quorum Technologies, Laughton, UK).

### 2.4. Proximate Composition Analysis of Black Glutinous Rice Crackers

Proximate composition of black glutinous rice was analyzed in triplicate using AOAC methods [22], and expressed on a dry basis (db). Moisture content was determined according to the AOAC method 925.10 using a hot-air oven (Binder FD115) at 105 ± 2 °C until constant weight was obtained. Crude protein was analyzed using the Kjeldahl method (AOAC method 954.01), and total nitrogen was converted to protein using a conversion factor of 6.25. Crude fat was determined following the AOAC method 920.39 using an automatic Soxhlet extraction system (E-816, BÜCHI Labortechnik AG, Flawil, Switzerland) with petroleum ether as the extraction solvent. Crude fiber was measured according to the AOAC method 962.09 by sequential digestion with 1.25% (*w*/*v*) sulfuric acid and 1.25% (*w*/*v*) potassium hydroxide using a Fibertec system (Fibertec 2010, FOSS, Hillerød, Denmark). Ash content was determined according to the AOAC method 923.03 by incinerating the samples in a muffle furnace (AAF11-18, Carbolite, Hope Valley, UK) at 550 °C. Carbohydrate was calculated by difference, and energy was estimated using Equation (3).

(3) $$Energy\;(Kcal/100\;g)=\left(fat\times 9\right)+\left(protein\times 4\right)+\left(carbohydrate\times 4\right)+(fiber\times 2)$$

### 2.5. Antioxidant Compounds and Activity Determination of Black Glutinous Rice Crackers

#### 2.5.1. Preparation of the Sample

Ground rice cracker (0.4 g) was extracted with 5 mL acidified methanol (80:20 *v*/*v*, 1% HCl) at 100 rpm and 25 °C for 2 h, and then centrifuged at 4000 rpm for 20 min. The supernatant was collected and refrigerated for same-day analysis of phenolic, flavonoid, anthocyanin contents, and DPPH activity [23].

#### 2.5.2. Determination of Total Phenolic Content (TPC)

Methanolic extract (0.2 mL) was mixed with 1.8 mL diluted Folin–Ciocalteu reagent, followed by 1.8 mL of 6% Na_2_CO_3_ after 5 min. The mixture was vortexed and incubated in the dark at room temperature for 90 min. Absorbance was measured at 725 nm. Results were expressed as mg gallic acid equivalents per g dry basis (mg GAE/g) using a gallic acid calibration curve [24].

#### 2.5.3. Determination of Total Flavonoid Content (TFC)

Methanolic extract (500 µL) was mixed with 3.0 mL deionized water and 5% NaNO_2_, followed by 10% AlCl_3_ after 6 min. After 5 min, 1 mL of 1 M NaOH was added, and the mixture was incubated in the dark at room temperature for 30 min. Absorbance was measured at 510 nm. TFC was expressed as mg quercetin equivalents per g dry basis (mg QE/g) using a quercetin standard curve [25].

#### 2.5.4. Determination of Total Anthocyanin Content (TAC)

TAC was determined using the pH differential method. Methanolic extract (0.5 mL) was mixed separately with potassium chloride buffer (0.025 M, pH 1.0) and sodium acetate buffer (0.025 M, pH 4.5), followed by vortexing and standing for 15 min. Absorbance was measured at 515 and 700 nm against an acidified ethanol blank. TAC was calculated based on the cyanidin-3-glucoside content using the Lambert–Beer Law as shown in Equation (4).

(4) $$TAC=\frac{A\times M_{W}\times DF\times 10^{3}}{\varepsilon \times L\times m}$$

where A is the difference between absorbance (A_515_–A_700_)_pH 1.0_ and (A_515_–A_700_)_pH 4.5_, M_W_ is the molecular weight of cyanidin-3-glucoside (449.2 g/mol), DF is the dilution factor, ε is the molar absorptivity coefficient (25,965 mol/L·cm), L is the cuvette optical pathlength (1 cm), and m is the weight of the sample (g). TAC was expressed as µg cyanidin-3-glucoside equivalent per g dry basis (µg CyE/g) [26].

#### 2.5.5. Determination of Antioxidant Activity

Methanolic extract (100 μL) was mixed with 2.9 mL DPPH solution (100 μM) and incubated in the dark at room temperature for 30 min. Absorbance was measured at 515 nm against a methanol blank. The DPPH radical scavenging activity was expressed as mg Trolox equivalent per g dry basis (mg TE/g) using a Trolox standard curve [27].

### 2.6. Optimization of Vacuum Frying Conditions

Regression models describing the relationships between independent variables and response parameters were developed using the least squares method. The adequacy of the fitted models was evaluated based on the coefficient of determination (R^2^) and analysis of variance (ANOVA). Experimental data were fitted to polynomial equations to identify the optimal vacuum frying conditions. In this study, the dependent variables included cooking yields (Y_1_), expansion ratio in thickness (Y_2_), bulk density (Y_3_), hardness (Y_4_), color change (Y_5_), water activity (Y_6_), moisture content (Y_7_), crude fat (Y_8_), TPC (Y_9_), and DPPH (Y_10_). The regression coefficients (β) were calculated by fitting the experimental data to a polynomial model as shown in Equation (5) [28].

(5) $$Yi=\beta_{0}+\sum \beta_{i}X_{i}+\sum \beta_{j}X_{j}+\sum \beta_{ij}X_{i}X_{j\;}+\sum \beta_{ii}X_{i}^{2}+\sum \beta_{jj}X_{j}^{2}$$

where Y_i_ is the response variable (Y_1_–Y_10_); X_i_ and X_j_ are the frying temperature and frying time, respectively. β_0,_ β_i_,β_j_, β_ij_, β_ii_, and β_ij_ are the regression coefficients.

The adequacy of the polynomial model to the responses was determined by the *p*-value of the model, the coefficient of determination (R^2^), the coefficient of adjustment (R^2^-adj), and the coefficient of prediction (R^2^-predicted). Based on the models, vacuum frying parameters were optimized. Then, three additional experiments were conducted to validate the optimum frying condition. The relative error was calculated to determine the predictability. In addition, the quality of rice crackers processed at the optimal vacuum frying condition was compared with that of crackers produced by atmospheric frying.

### 2.7. Statistical Analysis

All experiments were performed in triplicate, and results were expressed as mean ± standard deviation (SD). Two-way ANOVA was used to assess the effects of frying parameters on the physicochemical properties of rice crackers, followed by Duncan’s post hoc test at a 95% confidence level. Statistical analyses were conducted using SPSS (version 17.0). Optimization of vacuum frying conditions was performed using response surface methodology (RSM) with Design-Expert 13 software.

## 3. Results and Discussion

### 3.1. Effect of Vacuum Frying Parameters on the Physical Quality of Black Glutinous Rice Crackers

The physical properties of black glutinous rice crackers obtained from vacuum frying are presented in Table 1. Frying temperature and time significantly (*p* < 0.05) affected cooking yield, hardness, water activity, and color. In addition, the interaction between temperature and time significantly (*p* < 0.05) influenced hardness and water activity.

Frying at 80 °C yielded rice crackers of 83.37–86.15%. Increasing the frying temperature to 100 °C and 120 °C reduced the yield to 73.73–78.08% and 71.15–71.67%, respectively, reflecting enhanced water migration and greater weight loss during frying. This downward trend correlated with the decrease in water activity from 0.6241 to 0.0379 as frying temperature and time increased. These findings are consistent with the study by Lin et al. [29], which noted the cooking yield decreased as moisture evaporated rapidly at higher temperatures, as the rapid heat transfer from the oil to the product accelerates water migration and surface evaporation, thereby reducing final product mass and lowering yield. Fundamentally, frying reduces water activity as part of the dehydration process, making snacks microbiologically stable and structurally altered [30]. Similarly, in vacuum-fried carrot chips, increasing frying temperatures from 60 °C to 100 °C increased moisture removal, which was directly associated with lower water activity [31].

Hardness was significantly (*p* ≤ 0.05) affected by both frying temperature and time. For instance, frying at a low temperature (80 °C) for a short time (10 min) produced hard rice crackers (23.54 ± 3.57 N). Increasing the frying time to 20 min significantly (*p* ≤ 0.05) reduced hardness to 16.82 ± 3.13 N. Hardness further decreased to 4.19 ± 1.24 N when the frying temperature was raised to 120 °C for 20 min. This was likely associated with starch gelatinization and solubilization of internal structures at high moisture during the early stage of frying [32]. At 80 °C, gelatinization may have occurred slowly, potentially causing the crackers to remain hard for a longer duration. Conversely, at 100 °C, faster gelatinization and weakened cell walls appeared to reduce hardness greatly. In addition, higher frying temperatures presumably accelerated moisture evaporation, generating pores and lowering mechanical strength. As the temperature increased, rapid moisture diffusion and evaporation likely produced more porous and expanded structures [33,34], and this increased porosity concurrently reduced hardness and increased the crispiness in fried products [35]. Hardness did not decrease further at 120 °C, suggesting that the majority of starch gelatinization and moisture-driven softening had already occurred below 100 °C. Therefore, by the time the temperature reached 120 °C, the internal structure may have reached a stabilized, expanded state. Similarly, previous studies reported that rice- and starch-based systems showed rapid hardness reduction during heating at 70–100 °C, with little additional change at higher temperatures once moisture became very low and the structure reached mechanical equilibrium [29,36].

**Figure 1:**
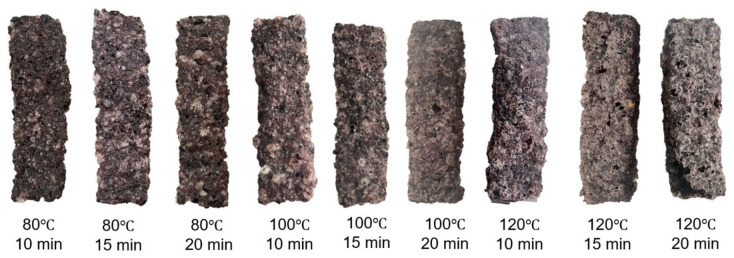
Appearance of black glutinous rice cracker under various vacuum frying conditions.

At 80 °C, the lightness (L*), redness (a*), and yellowness (b*) of vacuum-fried rice crackers remained stable regardless of frying time. This stability indicates that the low surface temperature was insufficient to cause major pigment degradation. Under vacuum conditions, the reduced boiling point and low oxygen level also limited browning and pigment breakdown, helping preserve the original color [37,38]. However, increasing both the frying temperature and time produced lighter crackers with lower redness and higher yellowness (Figure 1). Higher temperatures likely promoted Maillard reactions between reducing sugars and amino acids, forming brown melanoidin pigments commonly observed in vacuum-fried products [9,38]. Concurrently, thermal degradation of anthocyanins and other thermally sensitive pigments may have also contributed to lower a* and higher b* values through the formation of secondary yellow compounds. In addition, faster moisture evaporation could have created a drier, more porous crust that scattered light more strongly, resulting in higher L* values [39]. Consequently, the total color difference (ΔE) significantly (*p* ≤ 0.05) increased from 10.57 ± 1.43 to 14.97 ± 0.84 as frying temperature and time increased.

Considering the expansion ratio, frying temperature had a significant (*p* ≤ 0.05) effect on the expansion ratio in thickness. By increasing the temperature from 80 °C to 120 °C, the expansion ratio increased from 2.05–2.08 to 2.36–2.43. At 120 °C, but extending the frying time did not improve the expansion (Table 2). This may be explained by the high initial vapor pressure while the moisture content was still high, resulting in maximum expansion at the early stage of frying. As frying progressed, heat enhanced moisture loss, and then the vapor pressure presumably reduced while crust hardening restricted deformation [40,41]. As a result, the thickness did not expand as the frying time increased from 10 to 20 min at a high frying temperature (120 °C). However, neither frying temperature nor time significantly affected expansion in both width and length. This phenomenon can likely be explained by the rapid hardening of the outer layers of the rice crackers during the early stage of frying, which fixed their dimensions and prevented lateral expansion. Once the crust formed, further heating appeared ineffective at changing external dimensions, as both shrinkage and puffing were limited after the structure became rigid [40,42]. Although the expansion ratio was not improved, product weight loss increased with frying time. As a result, bulk density significantly (*p* ≤ 0.05) decreased from 0.63 to 0.49 g/cm^3^ at 80 °C, and from 0.47 to 0.38 g/cm^3^ at 100 °C. At the high frying temperature (120 °C), the bulk density of vacuum-fried rice crackers was the lowest.

**Figure 2:**
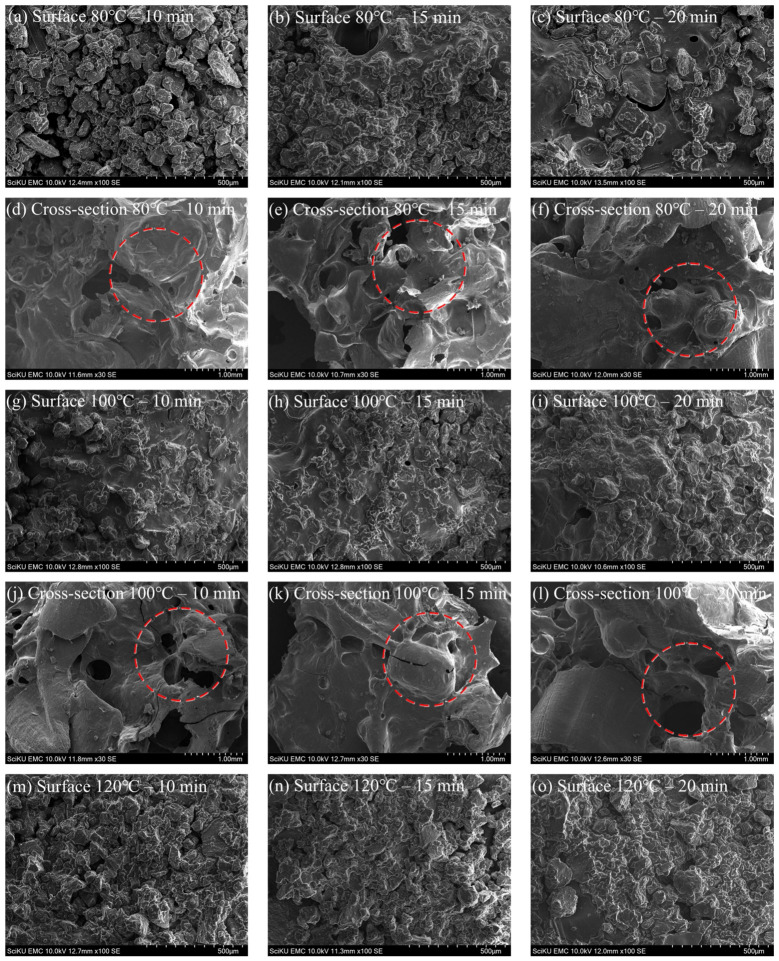

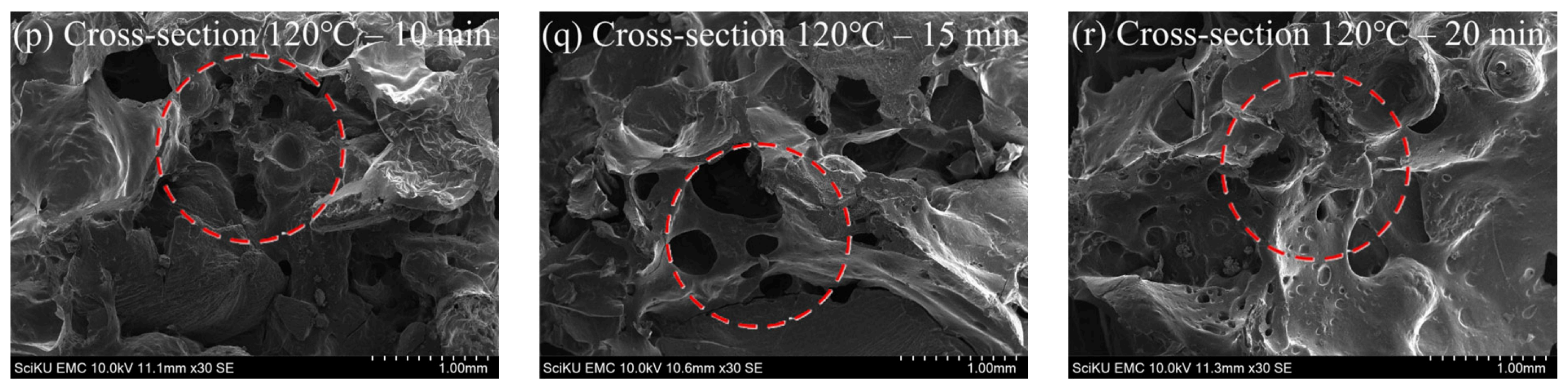
Scanning electron micrographs of the surface morphology (×100 magnification) and cross-section structure (×30 magnification) of rice crackers under vacuum frying conditions.

Regarding the microstructure of black glutinous rice crackers (Figure 2), frying at 80 °C for 10 min produced a relatively dense structure. SEM images of the surface showed a rough texture with some aggregated particles, while pores were not clearly visible. The cross-section also appeared compact with minimal voids. This was attributed to the probability of limited heat penetration, low moisture loss, and insufficient vapor pressure to form pores, resulting in minimal structural changes. When the frying time was extended to 15 min, increased heat transfer enhanced mass transfer and surface dehydration. Under these conditions, SEM images showed an irregular, fragmented surface with crack formation. The cross-section revealed partially ruptured cell walls and small voids, suggesting starch swelling and gelatinization with increased vapor pressure. Consequently, a preliminary porous structure began to form at this stage, which could potentially accommodate initial oil uptake. At 20 min, starch appeared extensively gelatinized, and moisture was largely removed. The surface became highly rough and discontinuous with visible pores and cracks, whereas the cross-section showed a well-developed, open, and highly permeable structure. This expanded structure contributed to a crisp texture but may also have facilitated oil absorption.

Increasing the frying temperature to 100–120 °C enhanced heat penetration and accelerated evaporation, leading to rapid surface dehydration and the formation of porous, expanded structures. These conditions also promoted faster internal structural changes, which were consistent with reports that higher frying temperatures accelerated porosity development and moisture diffusion, resulting in weaker and more open starch-based fried products [33,43]. Starch gelatinization quickly reached completion; therefore, after only 10 min, SEM images already showed a rough surface, large voids, and discontinuous structure. Extending frying time to 15–20 min further intensified structural collapse and pore expansion, suggesting increased crispiness with reduced hardness. However, the early formation of pores at 100–120 °C may have facilitated increased oil uptake by creating pathways for oil penetration, especially once the internal structure begins to collapse and the vapor pressure decreases, allowing oil to penetrate into the pore. In addition, porosity increased the surface roughness, which likely led to enhanced oil retention within the surface microstructure [44].

### 3.2. Effect of Vacuum Frying Parameters on Composition of Black Glutinous Rice Crackers

The nutritional composition of black glutinous rice crackers varied with vacuum frying conditions (Table 3). Frying temperature significantly affected moisture, ash, carbohydrate, and energy contents, while frying time significantly influenced moisture, protein, fat, and energy contents. In addition, the interaction between temperature and time significantly (*p* ≤ 0.05) affected moisture, fat, ash, and energy contents.

The moisture content ranged from 14.58 ± 1.46% at 80 °C–10 min to 1.96 ± 0.24% at 120 °C–20 min. It reduced as the frying temperature and time increased. Fat content was recorded between 22.14 ± 0.27% and 26.73 ± 0.52%. Higher fat from oil absorption was likely observed at long frying time (20 min), particularly frying at high temperature (120 °C). This trend aligns with the microstructure observed via SEM, suggesting that the increased frying temperature may have accelerated fast water migration from internal to outer layers. The resulting high vapor pressure could have promoted early pore development, high porosity, and roughness on the surface, thereby creating pathways that facilitated increased oil uptake to the rice crackers. Conversely, at shorter frying time, fat content remained comparatively low regardless of frying temperature. This finding highlights a significant (*p* ≤ 0.05) interaction effect between temperature and frying time on fat content.

Protein content fluctuated slightly from 6.63 ± 0.54% to 7.40 ± 0.36%, while fiber content remained stable between 2.40 ± 0.20% and 3.09 ± 1.10%. The ash content increased from 0.81 ± 0.03% at 80 °C to 1.13 ± 0.05% at 120 °C–20 min, primarily influenced by the increased frying temperature. This upward trend was likely driven by the progressive concentration of non-volatile minerals resulting from substantial moisture loss during frying, which increased the relative proportion of the ash fraction. Total carbohydrate values were observed from 50.88 ± 2.10% to 63.55 ± 0.16%. Consequently, the total energy of black glutinous rice crackers increased significantly (*p* ≤ 0.05) across treatments, starting at 452.64 ± 4.14 kcal/100 g and reaching a maximum of 513.99 ± 2.26 kcal/100 g at 120 °C–20 min condition. Both carbohydrate and energy were significantly (*p* ≤ 0.05) affected by the interaction between time and temperature. A positive relationship was observed, where increasing both processing parameters led to a significant elevation in these values. This trend was primarily driven by the progressive dehydration of the product and the concomitant oil accumulation within the rice cracker matrix during frying. According to Chittapalo and Songsanandr [45], black glutinous rice crackers with Panang flavor contained 14.98% fat, 16.51% protein, 61.62% carbohydrates, and provided 469 kcal/100 g, which shares a comparable nutritional profile with the findings of the present study.

### 3.3. Effect of Vacuum Frying Parameters on Antioxidant Compounds and Activity of Black Glutinous Rice Crackers

The antioxidant properties of black glutinous rice crackers under various vacuum frying conditions are shown in Table 4, including total phenolic content (TPC), total flavonoid content (TFC), total anthocyanin content (TAC), and DPPH values.

Frying temperature was a key factor affecting TPC. Increasing the temperature from 80 to 120 °C significantly (*p* ≤ 0.05) raised TPC from 2.21–2.40 to 2.72–2.78 mg GAE/g. This upward trend may be attributed to a potential increase in the release of bound phenolics through cell-wall disruption and thermal cleavage, as well as the possible formation of Maillard-derived antioxidant compounds at elevated temperatures [46]. Similarly, previous studies reported increased TPC after high-temperature treatment in sweet corn, artichoke, beans, and various fruits and vegetables, hypothesizing that this occurs due to heat-induced phenolic release and newly formed antioxidant compounds [47,48].

In contrast, TFC remained relatively stable across all treatments, ranging from 1.19 ± 0.16 to 1.44 ± 0.29 mg QE/g, with no significant (*p* > 0.05) differences. Correspondingly, TAC ranged from 20.22 ± 4.40 to 26.09 ± 6.87 µg CyE/g, indicating no significant (*p* > 0.05) effect of temperature or time. The statistical stability of both TFC and TAC under vacuum frying conditions suggests that the lower operating temperature, combined with the reduced oxygen environment, may have partially shielded these fractions from drastic thermal oxidation. Therefore, changing the frying time from 10 to 20 min or raising the temperature from 80 to 120 °C within the tested vacuum range did not induce any further statistically significant alterations in TFC and TAC.

For antioxidant activity, DPPH values varied significantly (*p* ≤ 0.05), from 3.48 ± 0.03 mg TE/g at 80 °C for 10 min to 3.01 ± 0.01 mg TE/g at 120 °C for 20 min. Although TFC and TAC were not significantly affected by frying conditions, DPPH scavenging capacity was significantly influenced by the interaction of temperature and time. This net decrease in DPPH radical scavenging capacity, despite an apparent increase in TPC, highlights a complex chemical trade-off. While thermal processing may elevate total extractable phenolics and generate early-stage Maillard intermediates, these newly liberated or formed compounds might possess lower individual radical scavenging efficiencies against DPPH radical compared to the native, highly active free antioxidants that could have undergone minor, localized alterations not fully captured by the broad TAC assay [49]. Additionally, the antagonistic or synergistic interactions among the changing profile of free phenolics, Maillard reaction products, and the food matrix during frying may alter the overall antioxidant performance.

However, vacuum frying appeared to support the relative retention of bioactive compounds, compared to typical atmospheric frying, likely due to lower processing temperatures and the minimized presence of oxygen [50,51]. A previous study similarly noted a high retention (approximately 86%) of anthocyanins in purple sweet potato chips subjected to vacuum frying [52].

### 3.4. Optimization of Vacuum Frying Parameters for Black Glutinous Rice Crackers

The relationships between independent frying variables (X_1_ and X_2_) and selected responses (Y_1_–Y_10_) of black glutinous rice crackers were established using regression models. The regression coefficients for polynomial models are presented in Table 5. Statistical analysis indicated that the developed models were adequate, with R^2^ values ranging from 0.858 to 0.999, indicating that a large proportion of the variability in the responses could be explained within the tested design space. The adjusted R^2^ values (0.810–0.997) further supported the adequacy of the models. The predicted R^2^ values for most responses (0.624–0.992) indicated reasonable predictive performance, whereas the lower values obtained for crude fat and DPPH models suggested possible overfitting and limited predictability for those specific parameters. Nevertheless, the difference between the adjusted R^2^ and predicted R^2^ values was less than 0.2, so the models were considered suitable for describing and predicting trends strictly within the evaluated experimental ranges.

**Figure 3:**
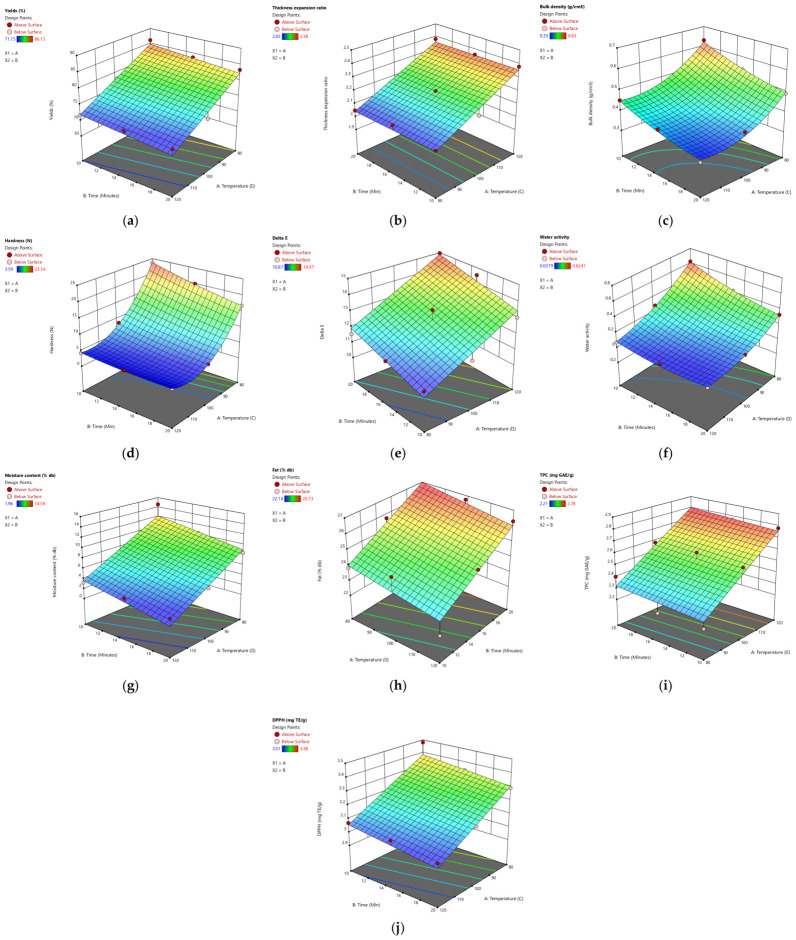
Three-dimensional responses surface of black glutinous rice cracker demonstrating effect of frying time and temperature on (**a**) cooking yields, (**b**) thickness expansion ratio, (**c**) bulk density, (**d**) hardness, (**e**) delta E, (**f**) water activity, (**g**) moisture content, (**h**) crude fat, (**i**) total phenolic content, and (**j**) DPPH.

The RSM plots (Figure 3) illustrate the effects of frying temperature (X_1_) and time (X_2_) on the responses (Y_1_–Y_10_) of black glutinous rice crackers. From Figure 3a,b,e, similar linear trends were observed, where cooking yield and thickness expansion ratio significantly increased with increasing temperature and longer frying time, while total color difference (ΔE) also progressively increased. From the results, higher yield was obtained at lower temperature and shorter time, which also minimized color change. In contrast, quadratic response behavior was observed for bulk density (Figure 3c) and hardness (Figure 3d), where the response surfaces formed characteristic “valley” or “U-shaped” patterns. From these figures, bulk density and hardness reached their minimum values at intermediate temperature and time, suggesting that the optimum crispiness was achieved through a balance between the rapid vapor-induced expansion of the gelatinized starch framework and structural collapse. In addition, moisture content (Figure 3g) and water activity (Figure 3f) decreased as the processing intensity increased, with both response surfaces exhibiting steep downward gradients toward the highest temperature and longest frying time, reflecting effective dehydration during vacuum frying. Interestingly, Figure 3i (TPC) and Figure 3j (DPPH) show positive linear trends, indicating that phenolic retention and antioxidant activity were maintained or enhanced at elevated frying temperatures. Overall, increasing temperature and time improved the expansion ratio while reducing moisture content, water activity, bulk density, hardness, and DPPH values. Total phenolic content increased mainly with temperature rather than time. In addition, high temperature combined with short frying time was effective in minimizing fat content in vacuum-fried rice crackers.

Based on the optimization objectives and constraints (Table 6), the optimal vacuum frying conditions for black glutinous rice crackers were estimated at 109.26 °C for 10 min, within the experimental range of 80–120 °C and 10–20 min. Under these conditions, the predicted responses included a cooking yield of 75.33%, a thickness expansion ratio of 2.27, and a bulk density of 0.45 g/cm^3^. Moisture content (5.92%db) and water activity (0.1626) were within desirable ranges, indicating storage stability. The optimized conditions also reduced undesirable attributes, resulting in low fat content (23.32%db), limited color change (ΔE = 12.65), and reduced hardness (4.83 N). In addition, the product exhibited potential for retaining antioxidant capacity, with total phenolic content of 2.63 mg GAE/g and DPPH of 3.14 mg TE/g. However, the optimal temperature of 109.26 °C was an estimated value from RSM; it could be rounded to 110 °C to practically set up for the subsequent experimental validation. Overall, vacuum frying at approximately 110 °C for 10 min showed potential to improve physical quality and phenolic content while reducing fat content and hardness in black glutinous rice crackers. The optimum vacuum frying condition identified in the present study was similar to those previously reported for other starch-based snack products. Esan et al. [38] reported the optimum vacuum frying for yellow-fleshed sweet potato chips at 108 °C for 9 min, producing healthier products with improved quality characteristics. Specifically, their optimized vacuum-fried samples contained approximately 60.42% less oil and retained a higher carotenoid content (6.01 mg/g) than the atmospheric fried product. In addition, Chintha et al. [52] observed the optimum vacuum frying of purple sweet potato chips at 105 °C for 7.08 min, which provided 86% retention of anthocyanins, 35.6% decline in oil content, and a reduced breaking force (0.69 N) relative to atmospheric deep-fried chips.

Three additional experiments were conducted to evaluate the empirical validity of the optimal conditions (Table 7). The experimental results showed reasonable agreement with the predicted values, particularly for yield and water activity, with low relative error (0.16% and 0.26%).

Although the hardness model exhibited a higher relative error (6.20%), the experimental data followed the predicted trend of decreasing hardness with increasing temperature, reaching a minimum of 4.16 N at 115 °C. The bioactive properties were also evaluated, as TPC and DPPH scavenging activity showed close agreement between experimental and predicted values, with relative errors of 1.02% and 1.55%, respectively. Bulk density remained stable at 0.45 g/cm^3^ across all conditions, which was consistent with the model predictions.

Overall, the low relative error supported the local applicability of the developed optimization models for the vacuum frying process for black glutinous rice crackers under the conditions evaluated. The quality of rice crackers produced under optimal vacuum frying conditions was compared with that of atmospheric deep-fat frying (Table 8). Vacuum frying significantly (*p* ≤ 0.05) reduced fat content from 37.31 ± 0.81% to 23.82 ± 0.73%. The vacuum-fried crackers also showed a greater thickness expansion ratio (2.26 ± 0.55) and lower hardness (5.10 ± 1.76 N) than the atmospheric-fried samples (16.27 ± 4.02 N). The lower oil absorption observed in vacuum-fried black glutinous rice crackers can be attributed to the combined effects of controlled moisture evaporation, pore development, and preservation of matrix integrity. Under vacuum frying conditions, water vaporizes at lower temperatures, allowing gentle vapor expansion within the gelatinized starch and promoting the formation of a stable porous structure while minimizing structural collapse from excessive thermal stress. Therefore, this preserved porous framework increases the mechanical stability of the pore walls (without collapse) and improves crispiness, compared with atmospheric frying.

Conversely, the color change (ΔE) and bulk density had no significant (*p* > 0.05) difference between treatments. In addition, the optimized vacuum-fried samples had higher total phenolic content (2.60 vs. 1.33 mg GAE/g), likely due to the lower temperature and reduced oxygen environment, which better preserved heat- and oxygen-sensitive phenolics. Overall, vacuum frying improved product quality under the parameters tested, resulting in 36.16% reduction in fat content, 95.49% higher TPC, 68.65% lower hardness, 10.78% greater thickness expansion, and 23.62% lower water activity, indicating a lighter, crispier product with better shelf-life stability than atmospheric frying.

## 4. Conclusions

Black glutinous rice demonstrated the potential to produce rice crackers with improved nutritional and physicochemical properties using vacuum frying. Compared to atmospheric deep frying, vacuum frying produced crackers with lower fat content and higher levels of bioactive compounds. Frying temperature and time significantly (*p* ≤ 0.05) influenced key quality attributes, particularly texture, water activity, and color, while protein, fiber, and flavonoid contents were not significantly (*p* > 0.05) affected. Higher temperatures and longer frying times enhanced crispiness and reduced water activity, supporting product stability. Optimization using RSM indicated optimal vacuum frying conditions at approximately 110 °C for 10 min. Under these conditions, the products exhibited a reduction in fat content, alongside improved texture and total phenolic content compared to atmospheric deep-fried samples. SEM analysis further supported these findings, showing the development of a more porous structure under vacuum frying. Overall, within the parameters evaluated, vacuum frying is an effective approach for producing healthier rice crackers from black glutinous rice, offering reduced oil uptake and enhanced functional properties.

## Figures and Tables

**Table 1:** Physical quality of black glutinous rice crackers under vacuum frying conditions.

Sample	Cooking Yield (%)	Hardness (N)	Water Activity	L*	a*	b*	∆E
80 °C–10 min	86.15 ± 3.20 ^a^	23.54 ± 3.57 ^a^	0.6241 ± 0.0079 ^a^	26.52 ± 2.31 ^c^	3.17 ± 0.32 ^cd^	1.40 ± 0.22 ^bcd^	10.87 ± 2.31 ^c^
80 °C–15 min	83.77 ± 2.84 ^a^	20.06 ± 3.40 ^b^	0.4285 ± 0.0048 ^b^	26.69 ± 2.40 ^c^	2.89 ± 0.52 ^d^	1.22 ± 0.45 ^cd^	11.02 ± 2.41 ^c^
80 °C–20 min	83.37 ± 4.41 ^a^	16.82 ± 3.13 ^c^	0.3283 ± 0.0013 ^c^	26.24 ± 1.41 ^c^	2.88 ± 0.44 ^d^	1.13 ± 0.33 ^d^	10.57 ± 1.43 ^c^
100 °C–10 min	78.08 ± 1.63 ^b^	8.37 ± 2.45 ^d^	0.2782 ± 0.0043 ^d^	27.11 ± 1.87 ^c^	3.36 ± 0.60 ^bc^	1.41 ± 0.42 ^bcd^	11.48 ± 1.90 ^c^
100 °C–15 min	74.09 ± 2.68 ^bc^	5.26 ± 1.67 ^e^	0.1257 ± 0.0016 ^e^	28.94 ± 1.88 ^b^	3.24 ± 0.33 ^cd^	1.46 ± 0.24 ^bc^	13.28 ± 1.88 ^b^
100 °C–20 min	73.73 ± 3.14 ^bc^	4.30 ± 1.30 ^ef^	0.1133 ± 0.0093 ^f^	28.82 ± 0.93 ^b^	3.72 ± 0.15 ^a^	1.63 ± 0.19 ^b^	13.20 ± 0.94 ^b^
120 °C–10 min	71.67 ± 2.61 ^c^	4.19 ± 1.24 ^f^	0.0733 ± 0.0100 ^g^	28.66 ± 1.50 ^b^	3.63 ± 0.30 ^ab^	1.53 ± 0.26 ^b^	13.03 ± 1.52 ^b^
120 °C–15 min	71.53 ± 2.59 ^c^	3.85 ± 1.11 ^f^	0.0468 ± 0.0036 ^h^	30.16 ± 0.87 ^ab^	3.73 ± 0.23 ^a^	1.68 ± 0.22 ^b^	14.54 ± 0.88 ^ab^
120 °C–20 min	71.15 ± 2.68 ^c^	3.59 ± 0.95 ^f^	0.0379 ± 0.0048 ^h^	30.56 ± 0.82 ^a^	3.91 ± 0.22 ^a^	1.98 ± 0.26 ^a^	14.97 ± 0.84 ^a^
*p*-value	0.000 *	0.000 *	0.000 *	0.000 *	0.000 *	0.000 *	0.000 *
**Probability**							
Temperature	0.000 *	0.000 *	0.000 *	0.000 *	0.000 *	0.000 *	0.000 *
Time	0.134	0.000 *	0.000 *	0.011	0.076	0.156	0.012 *
Temperature × Time	0.735	0.000 *	0.000 *	0.218	0.035 *	0.007 *	0.211

Values are expressed as mean ± SD. Different lowercase letters (a–h) within each column indicate significant differences (*p* < 0.05). * Statistical significance (*p* < 0.05).

**Table 2:** Bulk density and expansion ratio of black glutinous rice crackers under vacuum frying conditions.

Sample	Bulk Density (g/cm^3^)	Expansion Ratio in Length	Expansion Ratio in Width	Expansion Ratio in Thickness
80 °C–10 min	0.63 ± 0.05 ^a^	1.09 ± 0.05 ^a^	1.12 ± 0.01 ^a^	2.05 ± 0.04 ^b^
80 °C–15 min	0.54 ± 0.05 ^b^	1.09 ± 0.03 ^a^	1.08 ± 0.06 ^a^	2.08 ± 0.16 ^b^
80 °C–20 min	0.49 ± 0.04 ^bc^	1.10 ± 0.04 ^a^	1.11 ± 0.05 ^a^	2.05 ± 0.14 ^b^
100 °C–10 min	0.47 ± 0.05 ^bcd^	1.08 ± 0.02 ^a^	1.08 ± 0.03 ^a^	2.15 ± 0.02 ^b^
100 °C–15 min	0.40 ± 0.01 ^def^	1.03 ± 0.02 ^a^	1.11 ± 0.03 ^a^	2.00 ± 0.09 ^b^
100 °C–20 min	0.38 ± 0.03 ^ef^	1.03 ± 0.05 ^a^	1.08 ± 0.05 ^a^	2.37 ± 0.18 ^a^
120 °C–10 min	0.45 ± 0.05 ^cde^	1.08 ± 0.02 ^a^	1.14 ± 0.06 ^a^	2.43 ± 0.19 ^a^
120 °C–15 min	0.39 ± 0.01 ^ef^	1.07 ± 0.01 ^a^	1.13 ± 0.05 ^a^	2.36 ± 0.10 ^a^
120 °C–20 min	0.33 ± 0.05 ^f^	1.04 ± 0.03 ^a^	1.10 ± 0.04 ^a^	2.38 ± 0.06 ^a^
*p*-value	0.000 *	0.094	0.644	0.000 *
**Probability**				
Temperature	0.000 *	0.030	0.264	0.000 *
Time	0.000 *	0.200	0.704	0.191
Temperature × Time	0.689	0.370	0.657	0.113

Values are expressed as mean ± SD. Different lowercase letters (a–f) within each column indicate significant differences (*p* < 0.05). * Statistical significance (*p* < 0.05).

**Table 3:** Proximate composition of black glutinous rice crackers under different vacuum frying conditions.

	Parameters	Moisture (%db)	Fat (%db)	Protein (%db)	Fiber (%db)	Ash (%db)	Carbohydrate (%db)	Energy (kcal/100 g)
Conditions								
80 °C–10 min		14.58 ± 1.46 ^a^	23.84 ± 0.36 ^d^	7.40 ± 0.36 ^a^	2.49 ± 0.33 ^a^	0.81 ± 0.03 ^a^	50.88 ± 2.10 ^f^	452.64 ± 4.14 ^f^
80 °C–15 min		10.38 ± 0.54 ^b^	25.76 ± 0.60 ^b^	7.00 ± 0.19 ^abc^	2.79 ± 0.51 ^a^	0.80 ± 0.02 ^a^	53.26 ± 1.75 ^e^	478.50 ± 1.47 ^e^
80 °C–20 min		8.42 ± 0.43 ^c^	26.36 ± 0.48 ^ab^	6.77 ± 0.13 ^ab^	2.45 ± 0.20 ^a^	0.81 ± 0.02 ^a^	55.19 ± 0.41 ^e^	490.03 ± 3.50 ^d^
100 °C–10 min		6.24 ± 0.39 ^d^	24.50 ± 0.68 ^cd^	7.11 ± 0.14 ^abc^	2.40 ± 0.20 ^a^	0.85 ± 0.03 ^ab^	58.90 ± 1.29 ^cd^	489.33 ± 1.83 ^d^
100 °C–15 min		5.11 ± 0.31 ^e^	24.89 ± 0.55 ^c^	6.96 ± 0.29 ^abc^	2.45 ± 0.27 ^a^	0.87 ± 0.04 ^ab^	59.71 ± 1.02 ^cd^	495.61 ± 1.56 ^c^
100 °C–20 min		4.32 ± 0.17 ^e^	26.73 ± 0.52 ^a^	6.98 ± 0.13 ^abc^	3.09 ± 1.10 ^a^	0.89 ± 0.04 ^b^	57.99 ± 1.02 ^d^	506.61 ± 5.31 ^b^
120 °C–10 min		3.22 ± 0.36 ^f^	22.14 ± 0.27 ^e^	7.36 ± 0.14 ^a^	2.59 ± 0.48 ^a^	1.14 ± 0.04 ^a^	63.55 ± 0.16 ^a^	488.07 ± 1.06 ^d^
120 °C–15 min		2.75 ± 0.51 ^fg^	24.66 ± 0.37 ^cd^	6.63 ± 0.54 ^c^	2.75 ± 0.22 ^a^	1.13 ± 0.06 ^a^	62.09 ± 0.70 ^ab^	502.27 ± 4.05 ^b^
120 °C–20 min		1.96 ± 0.24 ^g^	26.32 ± 0.39 ^ab^	7.21 ± 0.09 ^ab^	2.63 ± 0.27 ^a^	1.13 ± 0.05 ^a^	60.75 ± 0.36 ^bc^	513.99 ± 2.26 ^a^
*p*-value		0.000 *	0.000 *	0.034 *	0.744	0.000 *	0.000 *	0.000 *
**Probability**								
Temperature		0.000 *	0.000 *	0.918	0.925	0.000 *	0.000 *	0.000 *
Time		0.000 *	0.000 *	0.009 *	0.591	0.880	0.570	0.000 *
Temperature × Time		0.000 *	0.003 *	0.085	0.460	0.841	0.000 *	0.000 *

Values are expressed as mean ± SD. Different lowercase letters (a–g) within each column indicate significant differences (*p* < 0.05). * Statistical significance (*p* < 0.05).

**Table 4:** Antioxidant compounds and activity of black glutinous rice crackers under vacuum frying conditions.

	Parameters	TPC (mg GAE/g)	TFC (mg QE/g)	TAC (µg CyE/g)	DPPH (mg TE/g)
Conditions					
80 °C–10 min		2.22 ± 0.21 ^c^	1.38 ± 0.40 ^a^	26.09 ± 6.87 ^a^	3.48 ± 0.03 ^a^
80 °C–15 min		2.21 ± 0.28 ^c^	1.25 ± 0.28 ^a^	22.55 ± 8.51 ^a^	3.34 ± 0.02 ^b^
80 °C–20 min		2.40 ± 0.21 ^bc^	1.44 ± 0.29 ^a^	23.30 ± 7.16 ^a^	3.29 ± 0.02 ^c^
100 °C–10 min		2.57 ± 0.04 ^ab^	1.40 ± 0.27 ^a^	20.22 ± 4.40 ^a^	3.12 ± 0.05 ^d^
100 °C–15 min		2.59 ± 0.27 ^ab^	1.20 ± 0.09 ^a^	23.62 ± 3.39 ^a^	3.13 ± 0.02 ^d^
100 °C–20 min		2.58 ± 0.10 ^ab^	1.36 ± 0.32 ^a^	23.16 ± 6.77 ^a^	3.13 ± 0.02 ^d^
120 °C–10 min		2.78 ± 0.01 ^a^	1.41 ± 0.18 ^a^	24.39 ± 3.84 ^a^	3.07 ± 0.02 ^e^
120 °C–15 min		2.77 ± 0.17 ^a^	1.43 ± 0.16 ^a^	22.67 ± 5.68 ^a^	3.05 ± 0.03 ^e^
120 °C–20 min		2.72 ± 0.12 ^ab^	1.19 ± 0.16 ^a^	22.42 ± 3.70 ^a^	3.01 ± 0.01 ^f^
*p*-value		0.005 *	0.891	0.723	0.000 *
**Probability**					
Temperature		0.001 *	0.781	0.589	0.000 *
Time		0.828	0.844	0.906	0.000 *
Temperature × Time		0.761	0.634	0.409	0.000 *

Values are expressed as mean ± SD. Different lowercase letters (a–f) within each column indicate significant differences (*p* < 0.05). * Statistical significance (*p* < 0.05).

**Table 5:** Regression coefficients and analysis of variance of the first- and second-order polynomials of black glutinous rice crackers under vacuum frying conditions.

Parameter	Y_1_	Y_2_	Y_3_	Y_4_	Y_5_	Y_6_	Y_7_	Y_8_	Y_9_	Y_10_
**Model (** ***p*** **-value)**	0.000	0.000	0.004	0.000	0.000	0.001	0.001	0.003	0.001	0.002
**R^2^**	0.928	0.951	0.989	0.999	0.930	0.996	0.901	0.858	0.920	0.874
Intercept	113.34	1.417	2.575	229.502	3.077	4.870	32.209	22.924	1.273	4.117
**Linear**										
Temperature (X_1_)	−0.325	0.008	−0.032	−3.652	0.074	−0.062	−0.212	−0.024	0.012	−0.008
Time (X_2_)	−0.255	0.000	−0.035	−2.404	0.145	−0.132	−0.311	0.298	0.004	−0.008
**Quadratic**										
Temp × Temp (X_1_^2^)	-	-	0.000	0.015	-	0.000	-	-	-	-
Time × Time (X_2_^2^)	-	-	0.001	0.017	-	0.002	-	-	-	-
**Interactions**										
Temp × Time (X_1_X_2_)	-	-	0.000	0.015	-	0.001	-	-	-	-
**Adjusted R^2^**	0.903	0.935	0.970	0.997	0.906	0.990	0.868	0.810	0.893	0.832
**Predicted R^2^**	0.851	0.897	0.869	0.992	0.843	0.963	0.734	0.624	0.793	0.693

X_1_: frying temperature (°C); X_2_: frying time (min); Y_1_: cooking yields (%); Y_2_: expansion ratio in thickness; Y_3_: bulk density (g/cm^3^); Y_4_: hardness (N); Y_5_: delta E; Y_6_: water activity; Y_7_: moisture content (%db); Y_8_: crude fat (%db); Y_9_: total phenolic content (mg GAE/g); Y_10_: DPPH (mg TE/g).

**Table 6:** Optimization of vacuum frying conditions for black glutinous rice crackers.

Variable	Objective/ Constrain	Experimental Range		Optimum Condition
		Min	Max	
**Independent variable**				
Temperature (°C)	Optimum	80	120	109.26
Time (minutes)	Optimum	10	20	10.00
**Response variable**				
Cooking yield (%)	Range	71.15	86.15	75.33
Thickness expansion ratio	Maximize	2.05	2.38	2.27
Bulk density (g/cm^3^)	Minimize	0.33	0.63	0.45
Moisture content (%db)	Range	1.97	14.58	5.92
Fat (%db)	Minimize	22.14	26.73	23.32
∆E	Minimize	10.87	14.97	12.65
Water activity	Range	0.0379	0.6241	0.1626
Hardness (N)	Minimize	3.59	23.54	4.83
TPC (mg GAE/g)	Maximize	2.21	2.78	2.63
DPPH (mg TE/g)	Maximize	3.01	3.48	3.14

**Table 7:** Comparison between predicted and actual values of response variables at optimal vacuum frying conditions.

Temperature (°C)	105		110		115		
Time (minutes)	10		10		10		
Response variable	Predict	Experiment	Predict	Experiment	Predict	Experiment	%Relative error
Cooking yield (%)	76.71	76.50	75.09	75.00	73.47	73.40	0.16
Thickness expansion ratio	2.24	2.25	2.28	2.26	2.32	2.27	1.18
Bulk density (g/cm^3^)	0.46	0.45	0.45	0.45	0.45	0.45	0.74
Moisture content (%db)	6.83	6.47	5.77	5.91	4.71	4.46	4.51
Fat (%db)	23.42	23.51	23.30	23.82	23.18	23.98	1.97
∆E	12.33	12.02	12.70	12.36	13.07	12.58	3.07
Water activity	0.2072	0.2062	0.1557	0.1559	0.1148	0.1146	0.26
Texture (N)	5.97	5.40	4.69	5.10	4.16	4.16	6.20
TPC (mg GAE/g)	2.58	2.60	2.64	2.60	2.70	2.68	1.02
DPPH (mg TE/g)	3.18	3.26	3.14	3.20	3.10	3.11	1.55

**Table 8:** Comparison of rice crackers produced by optimized vacuum frying and atmospheric frying.

Response Variable	Atmospheric Fried Rice Crackers	Optimum Vacuum-Fried Rice Crackers
Cooking yield (%)	94.92 ± 2.89 ^a^	75.00 ± 3.14 ^b^
Thickness expansion	2.04 ± 0.09 ^b^	2.26 ± 0.55 ^a^
Bulk density (g/cm^3^)	0.42 ± 0.05 ^a^	0.45 ± 0.01 ^a^
Moisture content (%db)	6.42 ± 0.25 ^a^	5.91 ± 0.17 ^b^
Fat (%db)	37.31 ± 0.81 ^a^	23.82 ± 0.73 ^b^
∆E	11.15 ± 2.34 ^a^	12.36 ± 0.35 ^a^
Water activity	0.2041 ± 0.0024 ^a^	0.1559 ± 0.0009 ^b^
Texture (N)	16.27 ± 4.02 ^a^	5.10 ± 1.76 ^b^
TPC (mg GAE/g)	1.33 ± 0.18 ^b^	2.60 ± 0.34 ^a^
DPPH (mg TE/g)	2.87 ± 0.03 ^b^	3.20 ± 0.09 ^a^

Values are expressed as mean ± SD. Different lowercase letters (a, b) within each row indicate significant differences (*p* < 0.05).

## Data Availability

The original contributions presented in this study are included in the article. Further inquiries can be directed to the corresponding author.
